# Targets for future clinical trials in Huntington's disease: What's in the pipeline?

**DOI:** 10.1002/mds.26007

**Published:** 2014-08-25

**Authors:** Edward J Wild, Sarah J Tabrizi

**Affiliations:** 1 Department of Neurodegenerative Disease, UCL Institute of Neurology, National Hospital for Neurology & NeurosurgeryQueen Square, London, WC1N 3BG, UK

**Keywords:** gene silencing, glial cells, HDAC inhibition, Huntington's disease, kynurenine monooxygenase, MAPK, phosphodiesterase inhibition, therapeutics

## Abstract

The known genetic cause of Huntington's disease (HD) has fueled considerable progress in understanding its pathobiology and the development of therapeutic approaches aimed at correcting specific changes linked to the causative mutation. Among the most promising is reducing expression of mutant huntingtin protein (mHTT) with RNA interference or antisense oligonucleotides; human trials are now being planned. Zinc-finger transcriptional repression is another innovative method to reduce mHTT expression. Modulation of mHTT phosphorylation, chaperone upregulation, and autophagy enhancement represent attempts to alter cellular homeostasis to favor removal of mHTT. Inhibition of histone deacetylases (HDACs) remains of interest; recent work affirms HDAC4 as a target but questions the assumed centrality of its catalytic activity in HD. Phosphodiesterase inhibition, aimed at restoring synaptic function, has progressed rapidly to human trials. Deranged cellular signaling provides several tractable targets, but specificity and complexity are challenges. Restoring neurotrophic support in HD remains a key potential therapeutic approach. with several approaches being pursued, including brain-derived neurotrophic factor (BDNF) mimesis through tyrosine receptor kinase B (TrkB) agonism and monoclonal antibodies. An increasing understanding of the role of glial cells in HD has led to several new therapeutic avenues, including kynurenine monooxygenase inhibition, immunomodulation by laquinimod, CB2 agonism, and others. The complex metabolic derangements in HD remain under study, but no clear therapeutic strategy has yet emerged. We conclude that many exciting therapeutics are progressing through the development pipeline, and combining a better understanding of HD biology in human patients, with concerted medicinal chemistry efforts, will be crucial for bringing about an era of effective therapies.

Huntington's disease (HD) is characterized by a number of certainties: It is inherited, fully penetrant, neurodegenerative, progressive, fatal, and caused by CAG repeat expansions in the gene encoding huntingtin. So far, another certainty has been the failure of every attempt to prevent or slow its progression in patients and mutation carriers.^1^ However, the known cause of HD and our ever-increasing understanding of the events that connect the mutation to the clinical features of the disease continue to inspire confidence that one or more dysfunctions leading to HD will prove tractable. Here we review those therapeutic targets in the pipeline, borne from our understanding of the diverse effects of the HD mutation, that we consider most likely to give rise to viable treatments that may reach clinical trials in the foreseeable future. Figure 1 gives an overview of the targets we discuss, and these are summarized in Table1.

**FIG 1:**
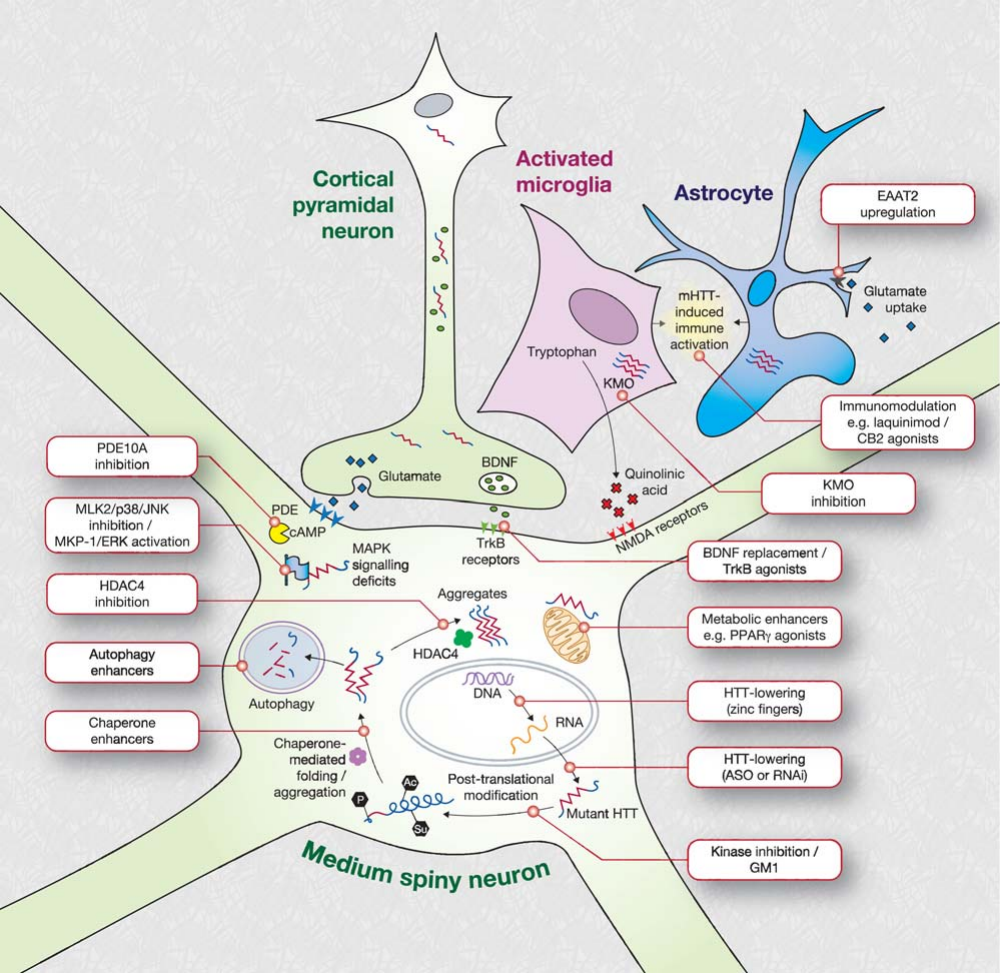
Schematic depicting current priority preclinical therapeutic targets under investigation for Huntington's disease. HTT, huntingtin; KMO, kynurenine monooxygenase; NMDA, N-methyl-D-aspartate; PDE, phosphodiesterase; BDNF, brain-derived neurotrophic factor; HDAC, histone deacetylase; Trk, tropomyosin-related kinase. Adapted from Ross et al.^36^

**TABLE 1:** Summary of current priority preclinical therapeutic targets in Huntington's disease^a^

Mechanism/Target	Compound	Tested in	Entity	Ref
HTT lowering by RNAi	Allele-nonspecific siRNA, intrastriatal convection-enhanced delivery	NHP	Alnylam Inc. / Medtronic, Inc.	21
	Allele-nonspecific siRNA, AAV delivery, intrastriatal injection	NHP	Medtronic Inc.	20
	Allele-nonspecific miRNA, AAV delivery, intrastriatal injection	NHP	U. Iowa	19
	Allele-nonspecific siRNA, cholesterol-conjugated, intrastriatal injection	Mouse	MGH / UMass / Alnylam Inc.	26
	Allele-specific single-stranded siRNA, intraventricular infusion	Mouse	UTSW / UCSD / Isis	28
HTT lowering by ASO	Allele-nonspecific ASO, intrathecal injection	NHP	Isis	22
	Allele-specific ASO, intrastriatal injection	Mouse	UBC / Isis	7
	Allele-specific CAG-targeted ASO	Cells	U. Leiden / Prosensa	23
HTT lowering by ZFP	Allele-specific ZFP transcriptional repressor, AAV delivery, intrastriatal injection	Mouse	EMBL/CRG SBRU	3
			Sangamo	3
Posttranslational modification	Kinase inhibition	Cells	McMaster U.	38
	Ganglioside GM1, intraventricular infusion	Mouse	McMaster U.	41
Chaperone enhancement	Genetic overexpression of HSJ1a	Mouse	KCL	44
	Recombinant ApiCCT1	Cells	UC Irvine / Stanford-	47
Autophagy enhancers	Trehalose, calpastatin, nicardipine, minoxidil	Mouse	Various	50
	Acetylation promoter (selisistat)	Human	Siena Biotech	52, 54
Aggregation prevention	HDAC4 genetic knockdown	Mouse	KCL	57
	Small molecule HDAC4 inhibition	Mouse	CHDI	61
Phosphodiesterase 10A inhibition	PF-2545920	Human	Pfizer	72
	OMS643762	Human	Omeros	^120^
MAPK cell signaling	JNK/p38 inhibition via MKP-1 overexpression	Mouse	EPFL	80
	MLK2 inhibition by CEP-1347	Mouse	UC Irvine	81
Neurotrophic support	TrkB agonists (7,8-DHF / 4′-DMA-7,8-DHF)	Mouse	Johns Hopkins	89
	TrkB agonist (LM22A-4)	Mouse	Stanford	90
	TrkB agonist (monoclonal antibody)	Cells	CHDI	91
	BDNF transcriptional activation	Zebrafish	U. Milano	92
	Cysteamine	Human	Raptor	94
KMO inhibition	JM6 / Ro-61-8048	Mouse	UCSF	102
	CHDI-340246	NHP	CHDI	103
Immunomodulation	Laquinimod	Human (MS)	Teva	108
	CB2 agonist (GW405833)	Mouse	UCSF	109
P2X7 antagonism	Brilliant blue G	Mouse	CSIC/UAM	110
Astrocytic glutamate uptake	EAAT2 promoter activation (ceftriaxone)	Mouse	U. Indiana	115

a ‘Tested in’ refers to most advanced model or organism in which successful target modulation has been demonstrated.

HTT, huntingtin protein; RNAi, RNA interference; siRNA, short interfering RNA; NHP, non-human primate; AAV, adeno-associated virus; miRNA, microRNA; MGH, Massachusetts General Hospital; ASO, antisense oligonucleotide; UBC, University of British Colombia; ZFP, zinc finger proteins; EMBL/CRG SBRU, European Molecular Biology Laboratory Systems Biology Research Unit; HSJ1a, Homo sapiens J domain protein 1a; ApiCCT1, apical domain of chaperonin containing T-complex protein-1/T-complex protein-1 ring subunit CCT1; KCL, King's College London; MAPK, mitogen-activated protein kinase; JNK, c-Jun terminal kinases; MKP-1, MAP kinase phosphatase 1; EPFL, École polytechnique fédérale de Lausanne; TrkB, tropomyosin-related kinase B; BDNF, brain-derived neurotrophic factor; UCSF, University of California San Francisco; KMO, kynurenine monooxygenase; MS, multiple sclerosis; CB2, cannabinoid Receptor 2; P2X7, purine receptor 2X7; CSIC, Consejo Superior de Investigaciones Científicas; UAM, Universidad Autónoma de Madrid; EAAT2, excitatory amino acid transporter 2.

## Reducing Huntingtin Expression

In contrast to other prevalent neurodegenerative disorders, the known genetic cause of HD allows the known pathogenic entity, mutant huntingtin protein (mHTT), to be targeted with certainty. Lowering expression of mHTT at the level of DNA (transcription) or RNA (translation) ought to reduce all of the downstream deleterious effects of the protein that lead to the manifestations of HD. Such strategies are sometimes known as “gene silencing”—somewhat misleadingly, because no approach is expected to stop mHTT expression altogether—or “huntingtin lowering” or “huntingtin suppression”. These approaches aimed at reducing HTT expression are considered among the most promising emerging therapeutics to slow or prevent HD.^2^,^3^

Three broad approaches are under investigation to reduce mHTT expression: RNA interference (RNAi) using short interfering RNA (siRNA); translational repression using single-stranded DNA-based antisense oligonucleotides (ASOs); and transcriptional repression using zinc finger proteins (ZFPs).

Some of these approaches constitute “gene therapy”—namely, those delivered or expressed using viral technology, such as ZFPs or some RNAi methods, whereas central nervous system (CNS) delivery of antisense oligonucleotides or siRNAs is not gene therapy.

### Nucleotide-Based Silencing

**FIG 2:**
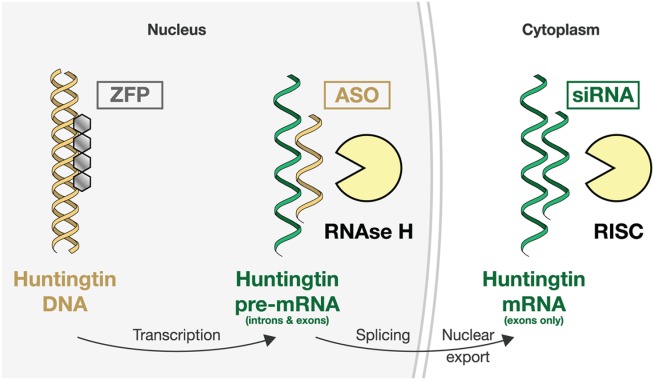
Schematic illustration of the three main approaches to lowering huntingtin expression. Zinc finger protein (ZFP) therapeutics aim to reduce transcription of the huntingtin gene. Translational repression can be attempted at the pre-mRNA level using DNA-based antisense oligonucleotides (ASOs) or on mature mRNA using short interfering RNA (siRNA) compounds. Different cellular mechanisms degrade the bound mRNA.^4^

RNA interference and ASO repression use synthetic modified nucleotide agents designed to bind to a chosen sequence in the HTT messenger RNA (mRNA), using Watson-Crick complementarity. Once bound, different cellular mRNA disposal mechanisms remove the HTT mRNA, resulting in reduced translation and lowered protein expression (Fig. 2).^2^,^4^

In RNAi, the drug molecule can be either an siRNA or a microRNA (miRNA) molecule. Degradation of siRNA-bound mRNA is performed by the RNA-induced silencing complex (RISC), which incorporates the RNAse enzyme argonaute. The ASOs are modified single-stranded DNA molecules, and ASO-bound mRNA is degraded by RNAse H (Fig. 2).^5^

Nucleotide-based gene silencing methods have advanced considerably in recent years and are approaching readiness for trials in human HD patients. Numerous successes have now been reported in rodent models, first with RNA-based drugs^6^ and more recently with ASOs.^7^ Most animal work has focused on nonselective silencing of both wild-type and mutant *HTT* alleles, and the first human trials will take this approach. Directly infused into the brain parenchyma or ventricles of HD model mice, these drugs appear capable of significantly reducing mRNA expression and HTT protein levels. This has been associated with not just slowing of the phenotypic progression of HD, but with substantial improvement in some manifestations having clinically significant counterparts in the human disease. For instance, intra-striatal injection of an adeno-associated virus (AAV2) vector expressing HTT-silencing miRNA in the YAC128 HD mouse model produced transduction of approximately 80% of the striatum, approximately 50% reduction in HTT mRNA, and a similar reduction in HTT protein; reduced mHTT aggregation; restored performance on a behavioral task modeling depression to near-wild-type levels; and showed no evidence of inflammation or neurotoxicity.^8^ The ASOs are no less successful: intraventricular infusion in three HD mouse models produced more than 60% reduction in HTT mRNA and more than 80% reduction in HTT protein; mHTT aggregate formation was delayed and motor performance improved with treatment. Strikingly, these improvements significantly outlasted both the presence of the ASO drug and the reduction in soluble protein,^9^ suggesting that dysfunctioning cells are able to recover from at least some deleterious effects of mHTT if expression of the protein is even transiently reduced, restoring the balance of damage and repair. Of course, whether this optimistic “huntingtin holiday” concept will translate into human patients for these therapeutics remains to be seen.^10^,^11^

In 2013, the first phase 1 human trial of an intrathecally delivered ASO, targeting superoxide dismutase 1 (SOD1) in familial amyotrophic lateral sclerosis, was completed without significant safety issues reported, paving the way for such trials with such agents in HD.^12^

### Potential Risks of Gene Silencing

Lowering huntingtin expression is not without its challenges. Safety is a major concern: both off-target effects and on-target lowering of wild-type HTT levels could produce unforeseen consequences in humans. The corollary of sustained benefit may be sustained adverse effects and the absence of an “off-switch,” particularly for gene therapy approaches such as ZFP, and viral delivery of siRNAs or miRNAs, but also for long-lasting drugs such as ASOs, is cause for proceeding with caution to human trials. A major unknown is the effect of lowering wild-type HTT in humans. HTT is clearly an important protein, because knocking out the gene is embryonic lethal in murine models,^13^ and conditional huntingtin knockout has been reported to produce neurodegeneration.^14^ Although transient long-acting ASO-induced HTT knockdown in wild-type BACHD mice by 75% produced no detectable behavioral or motor deficits,^9^ subtler effects could be missed in murine studies, and the effect of reducing wild-type HTT in human patients is unknown. However, we do know with certainty that mHTT expression causes HD; therefore, we hope that the benefits of lowering the toxic mHTT protein will significantly outweigh the potential side effects of lowering wild-type HTT.

Other safety concerns are generic to the molecules and delivery methodologies necessary to obtain translational repression in the CNS. The presence of synthetic oligonucleotides *per se*, some with backbone chemistries not seen in nature, could cause toxicity or neuroinflammation independently of their effects on gene expression.^15^ Although the safety and efficacy of viral vectors is improving, concerns remain around their possible immunogenicity, either on first dosing^16^ or as a limitation to the later administration of newer AAV-delivered compounds to patients dosed previously.

Future oligonucleotide-based gene silencing drugs are likely to be even more effective. Candidate nucleotide sequences have been optimized through rational design to maximize binding to HTT mRNA while minimizing off-target effects through binding to other mRNAs,^17^ whereas improvements in the nucleotide backbone chemistry promise improved specificity, potency, and stability.^18^

So far, the available safety data, especially from several recent nonhuman primate trials, are encouraging. In wild-type rhesus macaques, McBride and colleagues^19^ produced up to 45% sustained wild-type HTT reduction in the striatum using AAV-delivered shRNA without evidence of adverse effects; Grondin and colleagues^20^ demonstrated safety of AAV-mediated RNAi striatal wild-type HTT suppression over six months; Stiles and colleagues^21^ found 28 days' convection-enhanced siRNA delivery to be well tolerated; and ASOs infused into the lumbar cerebrospinal fluid produced distribution to the cortex and, to a lesser extent, some deep brain structures, without adverse effects.^22^

### Allele-Selective Silencing

One way to obviate the risk of WT HTT knockdown is to target the mutant allele selectively. Targeting the CAG repeat to achieve allele-selective knockdown is under investigation^23^ but carries a risk of off-target effects on other polyCAG-containing genes. Another strategy is to identify and target single-nucleotide polymorphisms (SNPs) on the mutant allele, an approach that may be able to provide allele-selective mHTT silencing for a certain percentage of HD mutation carriers, amounting to personalized genomic medicine in which individual subjects with the correct SNP genotype on the mutant allele may be treated.^7^,^24^ The existence of a few common haplotypes means some SNPs are overrepresented on alleles also bearing *HTT* expansions, suggesting that a small number of SNP-targeted drugs could provide allele-selective silencing for most individuals.^24^ However, targeting polymorphisms dramatically reduces the repertoire of possible RNA target sequences, increasing the chance of off-target effects; developing multiple agents, each targeting a different SNP, has significant regulatory, cost, and practical implications. Non-allele–selective approaches are much more likely to reach human trials sooner, because such agents are more advanced in the HD therapeutic pipeline; but both approaches are being actively developed.

### The Distribution Problem

The other major challenge is delivery and distribution of the HTT suppression agents in the CNS. Whereas in nonhuman primates, ASOs diffuse rather widely into the cortex when injected into the lumbar cerebrospinal fluid, their distribution is not universal, and in particular the striatum, affected prominently and early in HD, absorbs relatively little after lumbar injection.^22^ The siRNAs have even less natural diffusion and uptake, but this can be enhanced by a number of methods, including viral vectors, exosomes,^25^ cholesterol conjugation,^26^ convection-enhanced delivery, and novel conjugates of single-stranded siRNA compounds.^27^,^28^ Targeting both cortex and striatum using different delivery methods has been proposed to overcome these limitations, and we think this may be an important future therapeutic approach.^11^ This is supported by recent work demonstrating that reducing mHTT expression in both cortex and striatum is necessary for optimal suppression of relevant phenotypes in a murine model of HD.^29^ Meanwhile, the development of technologies such as the Roche “brain shuttle” raises the prospect of allowing CNS penetration by peripheral administration of potential therapeutic agents.^30^

### Zinc Fingers

Another exciting advance uses zinc finger protein repressors, which are transcription factor DNA-recognition motifs that can be designed to allow selective binding to specific DNA sequences, and fused to a transcriptional repressor domain (Fig. 2). Zinc-finger proteins (ZFPs) can repress protein production by reducing transcription.^31^ In theory, this combines the virtues of RNAi translational repression with the added advantages of obviating potential harm from toxicity of mHTT mRNA^32^ or from alternatively spliced HTT species that may lack the targeted mRNA sequence^33^—pathobiological mechanisms that have both been proposed in HD. Two groups have targeted the expanded CAG repeat that causes HD using ZFP-based compounds encoded by viral vectors. Serendipitously, the proximity of the CAG repeat to the 5' end of the *HTT* gene appears to confer considerable selectivity over other polyCAG-containing genes. The approach has so far demonstrated successful selective repression of mHTT and amelioration of motor manifestations in an HD mouse model, but it shares the delivery and distribution hurdles of other virally delivered HTT-lowering methods.^3^,^34^ The ability of ZFPs to target nuclease-induced DNA scission and repair raises the tantalizing prospect of true gene therapy for HD in which excessive CAGs are excised from the genomes of expansion carriers through “genome editing.”^35^

## Protein Homeostasis

Once expressed, mHTT interacts with hundreds of partners, undergoes dozens of post-translational modifications, forms intranuclear and cytoplasmic aggregates, and may be degraded through autophagy. The complex life of mHTT in cells offers a multitude of potential therapeutic targets.^1^,^36^ Prioritizing these is currently limited by a lack of understanding of the most toxic HTT species and the difficulty of modulating multifunctional targets.

Modulation of mHTT post-translational modification is appealing because it is carried out by enzymes that ought to be targetable by small molecule therapeutics (Fig. 1). Phosphorylation of N-terminal mHTT at serines 13 and 16 reduces its toxicity in vivo^37^ and affects its intracellular targeting,^38^ whereas phosphorylation at serine 421 restores the ability of mHTT to promote axonal vesicular transport and neurotrophic factor release.^39^ Small-molecule kinase inhibitors modulating N-terminal mHTT phosphorylation have been identified and are under investigation,^38^ but whether inhibitors that can specifically increase desirable phosphorylation of key residues, while avoiding harmful phosphorylation events elsewhere in mHTT, or other proteins, remains to be seen. The same is true of all potentially important post-translational modification. One striking recent report linked to post-translational modification concerns gangliosides—CNS-abundant glycosphingolipids with roles in membrane functioning and cell signaling that have been shown to be deficient in HD models.^40^ Chronic intraventricular infusion of ganglioside GM1 in YAC128 mice restored normal motor function and expression of the striatal marker DARPP32 and increased phosphorylation of HTT at serines 13 and 16.^41^ The mechanism of this intriguing result is unclear: It requires replication and further mechanistic study as a possible therapeutic avenue.

Although whether mHTT aggregates are neuroprotective, neurotoxic, or both remains unclear, disordered protein folding and aggregation are a potentially tractable hallmark of HD. Upregulation of chaperone proteins in an attempt to reduce harmful misfolding of mHTT has previously shown limited therapeutic potential in mammalian HD models.^42^,^43^ However, overexpression of HSJ1a in R6/2 mice was shown to reduce the formation of large nuclear aggregates and modestly delayed disease progression, surprisingly mediated by detergent-insoluble mHTT species that had already begun to aggregate.^44^ Another chaperone, TCP1-ring complex (TRiC), is known to suppress mHTT aggregation,^45^,^46^ and a recombinant subunit of TRiC, ApiCCT1, was recently shown to be able to enter cells, where it decreased the formation of visible inclusions and fibrillar oligomers and reduced mHTT-induced toxicity.^47^ Whether this is a viable therapeutic strategy remains to be seen, but an increased understanding of chaperone proteins and protein homeostasis is capable of generating novel, apparently tractable therapeutic targets.

Mutant huntingtin protein can be cleared by macroautophagy but impairs its own clearance through impaired cargo recognition.^48^ Enhancing autophagy through mammalian target of rapamycin (mTOR) inhibition by rapamycin improved phenotypes in fly and mouse models of HD,^49^ and a number of agents have shown similar effects in model systems,^50^ and upregulation of autophagy to clear mHTT is an important potential therapeutic strategy. A cellular imaging screen for autophagy enhancers revealed a candidate compound that was neuroprotective against mHTT and several related FDA-approved compounds with similar potential.^51^ Acetylation of mHTT targets it for degradation by autophagy^49^ and more generally, hypoacetylation of chromatin is a feature of HD, so promoting acetylation has been proposed as a therapeutic strategy. Selisistat, an inhibitor of the deacetylase sirtuin 1, was recently shown to suppress mHTT-induced pathology in *Drosophila*, mammalian HD cell models, and the R6/2 mouse, where it significantly improved survival and behavioral but not motor phenotype, and reduced aggregate formation.^52^ Whether this was accomplished through autophagy enhancement or another means is unclear, and the role of sirtuin 1 is controversial, its overexpression in HD mammalian models also having been reported as neuroprotective.^53^ A recent phase 1B clinical trial of selisistat in early HD demonstrated safety and tolerability.^54^

## Histone Deacetylase Inhibition

With the aim of correcting transcriptional dysregulation, histone deacetylase (HDAC) inhibitors have been under study for a number of years in HD. The HDACs are potent regulators of transcription through chromatin modification. The nonselective HDAC inhibitor suberoylanilide hydroxamic acid was shown to ameliorate the motor phenotype in R6/2 mice.^55^ Although compounds targeting HDAC1 and HDAC3 have been shown to ameliorate disease phenotypes in fly and cellular models,^56^ systematic work has shown HDAC4 to be the sole HDAC among 11 whose genetic knockdown ameliorates the HD phenotype in mouse models.^57^–^60^ HDAC4 inhibition has therefore been a focus for therapeutic development in HD, and potent, selective small-molecule inhibitors of its enzymatic function have been developed.^61^

Unexpectedly, though genetic HDAC4 knockdown improved neuropathology, synaptic function, motor phenotype, and lifespan in R6/2 mice, it did so without improving global transcriptional dysregulation. These double-transgenic animals show delayed cytoplasmic mHTT aggregation, and HDAC4 is now known to co-localize with cytoplasmic inclusions.^57^ This novel, cytoplasmic role for HDAC4 calls into question whether inhibition of its catalytic site is necessary or sufficient to recapitulate the strikingly favorable features of genetic HDAC4 knockdown. A reappraisal of the therapeutic effect of suberoylanilide hydroxamic acid in HD model mice revealed that it reduced HDAC4 level through increased degradation.^62^ Understanding and modulating the noncatalytic functions of HDAC4 is a focus of current study.

## Phosphodiesterase Inhibition

Altered synaptic plasticity is one potentially reversible cause of dysfunction in HD. Impairment of cyclic adenosine monophosphate (cAMP) signaling^63^ and dysregulation of gene transcription mediated by the cAMP response element (CRE)^64^ are established features of HD. Phosphodiesterase (PDE) 10A is almost exclusively expressed in the striatum,^65^ and its activity is intimately linked to the synaptic biology of medium spiny neurons whose death is a prominent feature of HD. PDE10A regulates cAMP and cyclic guanosine monophosphate signalling, synaptic plasticity and the response to cortical stimulation.^66^,^67^ PDE10A inhibition or genetic deletion produces numerous CRE–related gene expression changes^68^ and alterations in synaptic function^69^ suggested to be beneficial in schizophrenia and HD.^67^ In the R6/2 mouse, PDE10A inhibition with TP-10 ameliorated motor deficits, reduced striatal atrophy and increased brain-derived neurotrophic factor (BDNF) levels.^70^ Detailed study of PDE10A and its pharmacological inhibition is underway to validate it as a target in HD. One concern is that early death of striatal neurons might deplete PDE10A levels to the extent that the target is lost; however, PET imaging with the PDE10A radioligand [18F]-MNI-695 suggests that enzyme levels are sufficient even in manifest HD to expect a meaningful response.^71^ Clinical trials of PDE10A inhibition in HD patients are already underway, with motor and functional MRI endpoints.^72^ Other phosphodiesterases implicated in HD pathogenesis are also under study. PDE4 inhibition with rolipram, meanwhile, improved survival and ameliorated neuropathology and motor phenotypes in the R6/2 mouse.^73^

## Mitogen-Activated Protein Kinase Cell Signaling

Mitogen-activated protein kinase (MAPK) signaling is involved in the regulation of many cellular functions in response to a variety of stimuli. Abnormal MAPK signaling is a feature of HD; in particular, the MAPKs JNK (c-Jun terminal kinases), ERK (extracellular signal-regulated kinases), and p38, and the upstream kinase mixed lineage kinase 2 (MLK2), are overactive in HD.^74^–^76^ One effect of this may be impaired axonal transport, caused by JNK3-induced phosphorylation of kinesin-1.^77^ Additionally, p38 overactivity may contribute to NMDA-receptor–mediated excitotoxicity.^78^ Extracellular signal-regulated kinase overactivation is complex and may overall be protective in the presence of mHTT.^76^ Treatment of R6/2 mice with sodium butyrate was neuroprotective and extended survival; it also induced upregulation of MKP-1, a negative regulator of MAPK signaling.^79^ However, sodium butyrate likely acts via multiple mechanisms. Recently, specific overexpression of MKP-1 was shown to exert neuroprotective effects against mHTT through inhibition of JNK and p38.^80^ Pharmacological MLK2 inhibition reduced toxicity in several model systems and increased motor performance and BDNF levels in the R6/2 mouse.^81^ Small-molecule approaches to activate MKP-1 and ERK, or to inhibit MLK2, JNK, and p38, may be of value, but these pathways, their role in HD, and the optimal targets and means of modulating them are incompletely understood.

## Neurotrophic Factors

Depletion of BDNF is a well-established feature of the HD brain. Produced by cortical neurons, BDNF promotes neuronal growth, survival, and plasticity. It is particularly important for the survival of striatal neurons that are affected prominently in HD and may protect against excitotoxicity.^82^ Several mechanisms have been implicated in the depletion of BDNF in HD, including transcriptional dysregulation^83^ and reduced axonal transport.^84^ Restoration of BDNF levels, or those of related neurotrophins such as glial cell–derived neurotrophic factor (GDNF), is of interest, but the challenges of delivering a protein-based therapeutic to the CNS are considerable. Delivery of BDNF and GDNF using viral^85^ or stem-cell^86^ vehicles has shown some potential. Clinical trials in Parkinson's disease (PD) patients have demonstrated that intraparenchymal AAV-mediated delivery of the GDNF analog neurturin to the putamen is safe and well-tolerated but have yet to meet a primary efficacy endpoint.^87^ Postmortem analysis has confirmed successful induction and sustained expression of neurturin. A recently completed phase 2b trial has been reported as again meeting safety but not efficacy endpoints.^88^ One reason for efficacy failure of this approach may be the difficulty of identifying PD patients sufficiently early to intervene successfully. The monogenic, penetrant nature of HD perhaps makes it more amenable to this approach to neurotrophin delivery, because treatment could be initiated early in the disease or even before symptom onset.

BDNF acts principally through binding to TrkB receptors, and one approach to overcome the limitations of a protein-based therapeutic has been to develop small-molecule TrkB agonists. Several experimental compounds have now been tested in HD rodent models. Jiang and colleagues orally administered two presumed TrkB agonists (7,8-DHF and 4′-DMA-7,8-DHF) to N171-82Q mice and showed increased striatal TrkB phosphorylation, significantly improved motor function, increased lifespan, and reduced brain atrophy in treated animals.^89^ Simmons and colleagues^90^ demonstrated similar benefits from another TrkB agonist, LM22A-4, in the R6/2 and BACHD models, and additionally showed reduced intranuclear aggregation of mHTT in striatum and cortex.

However, Todd and colleagues^91^ compared 7,8-DHF, LM22A-4, and other reported small-molecule TrkB agonists. In contrast to previous reports, all tested compounds displayed a lack of TrkB agonism, no activation of relevant pathways, and no neuroprotection against mHTT in corticostriatal co-culture. However, two monoclonal antibodies were shown to agonize TrkB in a manner akin to BDNF and protected striatal neurons from mHTT-induced toxicity. Though challenging, the use of monoclonal antibodies as BDNF mimics warrants further study.^91^

An innovative approach to restoring neurotrophic support in HD is to target the transcriptional dysregulation that partly underlies the BDNF deficiency in HD. Abnormal repression of BDNF expression by the transcription factor REST/NRSF has been demonstrated in HD. Conforti and colleagues^92^ screened for compounds capable of inhibiting the formation of the REST-mSIN3 complex that is required for transcriptional repression. They identified a compound, C91, that increased BDNF mRNA levels in Htt-knockdown and mHTT-expressing zebrafish models.^92^ This novel approach is in its infancy but offers another avenue for rescuing the BDNF deficit in HD.

Finally, the FDA-approved compound cysteamine is thought to increase brain levels of BDNF by stimulating its release through an interaction with the heat-shock protein HSJ1b.^93^ A recent trial of cysteamine in HD patients has recently completed, but although a suggestion of motor improvement occurred in a subgroup analysis, the primary efficacy endpoint was not met; the full results of the trial, and its open-label extension, are awaited.^94^

## Modulation of Glial Activity

Although the clinical features of HD are undoubtedly driven by cell-autonomous effects of mHTT causing neuronal dysfunction and death, the role of non-neuronal cells in the pathobiology of HD is increasingly a focus for study and as a source of tractable therapeutic targets. Huntingtin is ubiquitously expressed, and glial cells may display cell-autonomous dysfunctions of their own,^95^ which may exacerbate an already precarious situation for neurons.

Excitotoxicity is a long-hypothesized contributor to neuronal dysfunction and death in HD. The earliest HD models were generated by intrastriatal injection of the excitotoxic NMDA agonist quinolinic acid (QA) in rodents.^96^ Quinolinic acid is an endogenous metabolite produced by the degradation of tryptophan by the kynurenine pathway. The enzyme kynurenine monooxygenase (KMO) is a key branchpoint in this pathway, and its activity determines the balance of QA and the neuroprotectant metabolites kynurenic acid (KA) and kynurenine. In the CNS, the kynurenine pathway is confined to microglial cells.^97^ QA levels are increased and KA levels decreased in post-mortem HD patient brain.^98^,^99^ A yeast genomic screen highlighted KMO as a leading therapeutic target,^100^ and subsequent work in drosophila has confirmed this.^101^ Zwilling and colleagues^102^ treated R6/2 HD model mice with a KMO inhibitor compound, JM6, and found increased brain levels of KA and decreased glutamate. Treated animals displayed improved survival, reduced loss of the synaptic marker synaptophysin, and a decrease in abnormal microglial activation. Neither JM6 nor its metabolites cross the blood–brain barrier, suggesting that its beneficial effects are mediated by peripheral KMO inhibition, producing beneficial effects for the CNS via the transit of an intermediate compound, possibly kynurenine.^102^ Subsequent work by Beconi and colleagues^120^ has questioned the status of JM6 as a KMO inhibitor, suggesting that the observed effects were likely attributable to contamination by the known KMO inhibitor Ro-61-8048; however, the status of KMO inhibition, peripherally or centrally, as a therapeutic target remains strong. Indeed a novel peripherally acting KMO inhibitor, CHDI-340246, has been reported to increase levels of kynurenine and KA in HD rodent models and the cerebrospinal fluid of nonhuman primates.^103^

Hyperactivity of the innate immune system, both centrally and peripherally, as a result of the cell-autonomous effects of mHTT in monocytes and microglia, and mediated by the nuclear factor kappa B (NFκB) pathway, is now an established pathogenic pathway in HD.^95^,^104^ Whether immunomodulation by any of the wide array of agents available is capable of preventing or reversing this detectable phenotype, and whether this will prove beneficial in patients, remains to be seen. Although its precise mechanism of action is unknown, the immunomodulator laquinimod reduces (NFκB) activation in astrocytes^105^ and may restore BDNF levels.^106^ Laquinimod also may act in part through the MAPK signaling pathway, reducing the phosphorylation of p38 and JNK,^107^ linking this compound to the cell signaling pathways discussed previously. Having demonstrated potential in multiple sclerosis,^108^ laquinimod's effects on tractable dysfunctions in HD are under investigation, and clinical trials in HD are planned.

CB2 cannabinoid receptors are expressed in microglia and peripheral immune cells; their activation is anti-inflammatory, and their levels are increased in postmortem HD brain. Genetic deletion of CB2 receptors was found to accelerate the phenotype in bacterial artificial chromosome HD (BACHD) mice, whereas treatment with the CB2 agonist GW405833 ameliorated it and prolonged survival. This effect was reversed by co-administration of a peripherally acting CB2 antagonist, suggesting again that peripheral immunomodulation may be capable of altering the CNS phenotype of HD.^109^

The microglial and neuronal P2X7 receptor is an adenosine triphosphate–gated ion channel that has been found to be overexpressed in synaptic terminals in HD.^110^ Extracellular adenosine triphosphate, acting on this receptor, stimulates synaptic dysregulation and neuronal death through apoptotic and nonapoptotic mechanisms^111^; in HD model mice, a P2X7 antagonist reduced apoptotic neuronal death, weight loss, and motor deficits.^110^ P2X7 in both neurons and microglia is under investigation as a potential therapeutic target.

Extracellular glutamate, which may contribute to excitotoxic neuronal death, is predominantly (90%) removed by excitatory amino acid transporter 2 (EAAT2), predominantly expressed in astrocytes.^112^ EAAT2 and its ortholog GLT1 show reduced expression in the R6/2 mouse and human HD brain^113^,^114^ although whether receptor deficiency is the cause of impaired glutamate in HD striatum is less clear.^115^ EAAT2 expression may be amenable to pharmacological modulation through activation of its promoter by the antibiotic ceftriaxone.^116^ In one study, ceftriaxone treatment increased overall receptor expression and ameliorated motor deficits in R6/2 mice.^115^ Whether EAAT2 in relevant cell populations is amenable to sustained pharmacological upregulation in HD, and whether this will be beneficial in patients, remains to be seen.

## Metabolism

Numerous alterations of cellular energetic mechanisms have been described in HD, albeit with inconsistent findings, especially comparing animal and human studies^117^; however, an association between energetic deficits and the length of the CAG triplet repeat presents a compelling case for a direct causation by the mutant gene.^118^ Human trials of several antioxidant molecules have not yielded any clear therapeutic success, however, and improvement of our understanding of HD-specific metabolic derangements is needed to develop more targeted therapeutics.^117^ Modulation of the metabolic transcriptional coactivator Peroxisome proliferator-activated receptor-gamma coactivator (PGC1α), perhaps through agonism of the nuclear receptor peroxisome proliferator-activated receptor gamma by rosiglitazone, has been reported as ameliorating motor deficits and increasing cortical BDNF in an HD mouse model, although apparently without effect on striatal pathology.^119^ A much improved understanding of the complex metabolic effects of mHTT is needed.

## Conclusion

As our understanding of the consequences of the HD mutation increases, so the range of tractable targets for therapeutic development broadens. While there are many potential targets, few are well-validated, and many single studies of purported success have yet to be replicated. Another problem is the shortcomings of our model systems and the failure, so far, of any agent that has been beneficial in an HD mouse model to prove so in human patients. Insights from studying patients are likely to be key to bridging this so-called “valley of death”: increasingly we are inclined and able to demonstrate relevant derangements in patients or patient-derived tissue before embarking on expensive and potentially hazardous clinical trials. Our understanding of therapeutic targets^36^ and our ability to prosecute them is better than ever, thanks in part to the increasingly prominent, concerted involvement of medicinal chemists in the field,^61^ and we anticipate an exciting era in the near future in which multiple agents, designed specifically to target the known pathobiology of HD, will enter clinical trials with a reasonable expectation of success.

## References

[1] Ross CA, Tabrizi SJ (2011). Huntington's disease: from molecular pathogenesis to clinical treatment. The Lancet Neurology.

[2] Magen I, Hornstein E (2014). Oligonucleotide-based therapy for neurodegenerative diseases. Brain Res.

[3] Garriga-Canut M, Agustín-Pavón C, Herrmann F (2012). Synthetic zinc finger repressors reduce mutant huntingtin expression in the brain of R6/2 mice. Proc Natl Acad Sci U S A.

[4] Bennett CF, Swayze EE (2010). RNA targeting therapeutics: molecular mechanisms of antisense oligonucleotides as a therapeutic platform. Annu Rev Pharmacol Toxicol.

[5] Martínez T, Wright N, López-Fraga M, Jiménez A, Pañeda C (2013). Silencing human genetic diseases with oligonucleotide-based therapies. Hum Genet.

[6] Harper SQ, Staber PD, He X (2005). RNA interference improves motor and neuropathological abnormalities in a Huntington's disease mouse model. Proc Natl Acad Sci U S A.

[7] Carroll JB, Warby SC, Southwell AL (2011). Potent and selective antisense oligonucleotides targeting single-nucleotide polymorphisms in the Huntington disease gene/allele-specific silencing of mutant huntingtin. Mol Ther.

[8] Stanek LM, Sardi SP, Mastis BM (2014). Silencing mutant huntingtin by AAV-mediated RNAi ameliorates disease manifestations in the YAC128 mouse model of Huntington's disease. Hum Gene Ther.

[9] Kordasiewicz HB, Stanek LM, Wancewicz EV (2012). Sustained therapeutic reversal of Huntington's disease by transient repression of huntingtin synthesis. Neuron.

[10] Lu X-H, Yang XW (2012). “Huntingtin holiday”: progress toward an antisense therapy for Huntington's disease. Neuron.

[11] Aronin N, Moore M (2012). Hunting down huntingtin. N Engl J Med.

[12] Miller TM, Pestronk A, David W (2013). An antisense oligonucleotide against SOD1 delivered intrathecally for patients with SOD1 familial amyotrophic lateral sclerosis: a phase 1, randomised, first-in-man study. Lancet Neurol.

[13] Nasir J, Floresco SB, O'Kusky JR (1995). Targeted disruption of the Huntington's disease gene results in embryonic lethality and behavioral and morphological changes in heterozygotes. Cell.

[14] Dragatsis I, Levine MS, Zeitlin S (2000). Inactivation of Hdh in the brain and testis results in progressive neurodegeneration and sterility in mice. Nat Genet.

[15] Marques JT, Williams BRG (2005). Activation of the mammalian immune system by siRNAs. Nat Biotech.

[16] Mingozzi F, High KA (2011). Immune responses to AAV in clinical trials. Curr Gene Ther.

[17] Boudreau RL, Spengler RM, Davidson BL (2011). Rational design of therapeutic siRNAs: minimizing off-targeting potential to improve the safety of RNAi therapy for Huntington's disease. Mol Ther.

[18] Dirin M, Winkler J (2013). Influence of diverse chemical modifications on the ADME characteristics and toxicology of antisense oligonucleotides. Exp Opin Biol Ther.

[19] McBride JL, Pitzer MR, Boudreau RL (2011). Preclinical safety of RNAi-mediated HTT suppression in the rhesus macaque as a potential therapy for Huntington's disease. Mol Ther.

[20] Grondin R, Kaytor MD, Ai Y (2012). Six-month partial suppression of Huntingtin is well tolerated in the adult rhesus striatum. Brain.

[21] Stiles DK, Zhang Z, Ge P (2012). Widespread suppression of huntingtin with convection-enhanced delivery of siRNA. Exp Neurol.

[22] Bennett CF (2011). Antisense oligonucleotide therapy for the treatment of Huntington's disease. World Congress on Huntington's Disease.

[23] Evers MM, Pepers BA, van Deutekom JC (2011). Targeting several CAG expansion diseases by a single antisense oligonucleotide. PloS One.

[24] Lombardi MS, Jaspers L, Spronkmans C (2009). A majority of Huntington's disease patients may be treatable by individualized allele-specific RNA interference. Exp Neurol.

[25] Alvarez-Erviti L, Seow Y, Yin H, Betts C, Lakhal S, Wood MJA (2011). Delivery of siRNA to the mouse brain by systemic injection of targeted exosomes. Nat Biotech.

[26] DiFiglia M, Sena-Esteves M, Chase K (2007). Therapeutic silencing of mutant huntingtin with siRNA attenuates striatal and cortical neuropathology and behavioral deficits. Proc Natl Acad Sci U S A.

[27] Lima WF, Prakash TP, Murray HM (2012). Single-stranded siRNAs activate RNAi in animals. Cell.

[28] Yu D, Pendergraff H, Liu J (2012). Single-stranded RNAs use RNAi to potently and allele-selectively inhibit mutant huntingtin expression. Cell.

[29] Wang N, Gray M, Lu X-H (2014). Neuronal targets for reducing mutant huntingtin expression to ameliorate disease in a mouse model of Huntington's disease. Nat Med.

[30] Niewoehner J, Bohrmann B, Collin L (2014). Increased brain penetration and potency of a therapeutic antibody using a monovalent molecular shuttle. Neuron.

[31] Papworth M, Kolasinska P, Minczuk M (2006). Designer zinc-finger proteins and their applications. Gene.

[32] Bañez-Coronel M, Porta S, Kagerbauer B (2012). A pathogenic mechanism in Huntington's disease involves small CAG-repeated RNAs with neurotoxic activity. PLoS Genetics.

[33] Sathasivam K, Neueder A, Gipson TA (2013). Aberrant splicing of HTT generates the pathogenic exon 1 protein in Huntington disease. Proc Natl Acad Sci U S A.

[34] Zeitler JRP, Froelich S, Yu Q (2013). Allele-specific repression of mutant Huntingtin expression by engineered zinc finger transcriptional repressors as a potential therapy for Huntington's disease. Society for Neuroscience Annual Meeting. San Diego, CA, USA.

[35] Li H, Haurigot V, Doyon Y (2011). In vivo genome editing restores haemostasis in a mouse model of haemophilia. Nature.

[36] Ross CA, Aylward EH, Wild EJ (2014). Huntington disease: natural history, biomarkers and prospects for therapeutics. Nat Rev Neurol.

[37] Gu X, Greiner ER, Mishra R (2009). Serines 13 and 16 are critical determinants of full-length human mutant huntingtin induced disease pathogenesis in HD mice. Neuron.

[38] Atwal RS, Desmond CR, Caron N (2011). Kinase inhibitors modulate huntingtin cell localization and toxicity. Nat Chem Biol.

[39] Zala D, Colin E, Rangone H, Liot G, Humbert S, Saudou F (2008). Phosphorylation of mutant huntingtin at S421 restores anterograde and retrograde transport in neurons. Hum Mol Genet.

[40] Maglione V, Marchi P, Di Pardo A (2010). Impaired ganglioside metabolism in Huntington's disease and neuroprotective role of GM1. J Neurosci.

[41] Di Pardo A, Maglione V, Alpaugh M (2012). Ganglioside GM1 induces phosphorylation of mutant huntingtin and restores normal motor behavior in Huntington disease mice. Proc Natl Acad Sci U S A.

[42] Hansson O, Nylandsted J, Castilho RF, Leist M, Jaattela M, Brundin P (2003). Overexpression of heat shock protein 70 in R6/2 Huntington's disease mice has only modest effects on disease progression. Brain Res.

[43] Hay DG, Sathasivam K, Tobaben S (2004). Progressive decrease in chaperone protein levels in a mouse model of Huntington's disease and induction of stress proteins as a therapeutic approach. Hum Mol Genet.

[44] Labbadia J, Novoselov SS, Bett JS (2012). Suppression of protein aggregation by chaperone modification of high molecular weight complexes. Brain.

[45] Kitamura A, Kubota H, Pack CG (2006). Cytosolic chaperonin prevents polyglutamine toxicity with altering the aggregation state. Nat Cell Biol.

[46] Tam S, Geller R, Spiess C, Frydman J (2006). The chaperonin TRiC controls polyglutamine aggregation and toxicity through subunit-specific interactions. Nat Cell Biol.

[47] Sontag EM, Joachimiak LA, Tan Z (2013). Exogenous delivery of chaperonin subunit fragment ApiCCT1 modulates mutant Huntingtin cellular phenotypes. Proc Natl Acad Sci U S A.

[48] Martinez-Vicente M, Talloczy Z, Wong E (2010). Cargo recognition failure is responsible for inefficient autophagy in Huntington's disease. Nat Neurosci.

[49] Ravikumar B, Vacher C, Berger Z (2004). Inhibition of mTOR induces autophagy and reduces toxicity of polyglutamine expansions in fly and mouse models of Huntington disease. Nat Genet.

[50] Renna M, Jimenez-Sanchez M, Sarkar S, Rubinsztein DC (2010). Chemical inducers of autophagy that enhance the clearance of mutant proteins in neurodegenerative diseases. J Biol Chem.

[51] Tsvetkov AS, Miller J, Arrasate M, Wong JS, Pleiss MA, Finkbeiner S (2010). A small-molecule scaffold induces autophagy in primary neurons and protects against toxicity in a Huntington disease model. Proc Natl Acad Sci U S A.

[52] Smith MR, Syed A, Lukacsovich T (2014). A potent and selective Sirtuin 1 inhibitor alleviates pathology in multiple animal and cell models of Huntington's disease. Hum Mol Genet.

[53] Jiang M, Wang J, Fu J (2012). Neuroprotective role of Sirt1 in mammalian models of Huntington's disease through activation of multiple Sirt1 targets. Nat Med.

[54] Reilmann R, Squitieri F, Priller J (2014). Safety and tolerability of selisistat for the treatment of Huntington's disease: results from a randomized, double-blind, placebo-controlled phase II trial (S47.004). Neurology.

[55] Hockly E, Richon VM, Woodman B (2003). Suberoylanilide hydroxamic acid, a histone deacetylase inhibitor, ameliorates motor deficits in a mouse model of Huntington's disease. Proc Natl Acad Sci U S A.

[56] Jia H, Pallos J, Jacques V (2012). Histone deacetylase (HDAC) inhibitors targeting HDAC3 and HDAC1 ameliorate polyglutamine-elicited phenotypes in model systems of Huntington's disease. Neurobiol Dis.

[57] Mielcarek M, Landles C, Weiss A (2013). HDAC4 reduction: a novel therapeutic strategy to target cytoplasmic huntingtin and ameliorate neurodegeneration. PLoS Biol.

[58] Moumné L, Campbell K, Howland D, Ouyang Y, Bates GP (2012). Genetic knock-down of HDAC3 does not modify disease-related phenotypes in a mouse model of Huntington's disease. PloS One.

[59] Bobrowska A, Paganetti P, Matthias P, Bates GP (2011). Hdac6 knock-out increases tubulin acetylation but does not modify disease progression in the R6/2 mouse model of Huntington's disease. PloS One.

[60] Benn CL, Butler R, Mariner L (2009). Genetic knock-down of HDAC7 does not ameliorate disease pathogenesis in the R6/2 mouse model of Huntington's disease. PLoS One.

[61] Dominguez C, Muñoz-Sanjuan I (2014). Foundation-directed therapeutic development in Huntington's disease. J Med Chem.

[62] Mielcarek M, Benn CL, Franklin SA (2011). SAHA decreases HDAC 2 and 4 levels in vivo and improves molecular phenotypes in the R6/2 mouse model of Huntington's disease. PLoS One.

[63] Gines S, Seong IS, Fossale E (2003). Specific progressive cAMP reduction implicates energy deficit in presymptomatic Huntington's disease knock-in mice. Hum Mol Genet.

[64] Sugars KL, Brown R, Cook LJ, Swartz J, Rubinsztein DC (2004). Decreased cAMP response element-mediated transcription: an early event in exon 1 and full-length cell models of Huntington's disease that contributes to polyglutamine pathogenesis. J Biol Chem.

[65] Coskran TM, Morton D, Menniti FS (2006). Immunohistochemical localization of phosphodiesterase 10A in multiple mammalian species. J Histochem Cytochem.

[66] Threlfell S, Sammut S, Menniti FS, Schmidt CJ, West AR (2009). Inhibition of phosphodiesterase 10A increases the responsiveness of striatal projection neurons to cortical stimulation. J Pharmacol Exp Ther.

[67] Threlfell S, West AR (2013). Modulation of striatal neuron activity by cyclic nucleotide signalling and phosphodiesterase inhibition. Basal Ganglia.

[68] Kleiman RJ, Kimmel LH, Bove SE (2011). Chronic suppression of phosphodiesterase 10A alters striatal expression of genes responsible for neurotransmitter synthesis, neurotransmission, and signaling pathways implicated in Huntington's disease. J Pharmacol Exp Ther.

[69] Piccart E, De Backer J-F, Gall D (2014). Genetic deletion of PDE10A selectively impairs incentive salience attribution and decreases medium spiny neuron excitability. Behav Brain Res.

[70] Giampà C, Laurenti D, Anzilotti S, Bernardi G, Menniti FS, Fusco FR (2010). Inhibition of the striatal specific phosphodiesterase PDE10A ameliorates striatal and cortical pathology in R6/2 mouse model of Huntington's disease. PLoS One.

[71] Zaleska M (2013). Advancing phosphodiesterase 10A (PDE10A) inhibitor from bench to clinic.

[72] (2014). National Institutes for Health. Study evaluating the safety, tolerability and brain function of 2 doses of PF-02545920 in subjects with early Huntington's disease.

[73] DeMarch Z, Giampà C, Patassini S, Bernardi G, Fusco FR (2008). Beneficial effects of rolipram in the R6/2 mouse model of Huntington's disease. Neurobiol Dis.

[74] Gianfriddo M, Melani A, Turchi D, Giovannini MG, Pedata F (2004). Adenosine and glutamate extracellular concentrations and mitogen-activated protein kinases in the striatum of Huntington transgenic mice: selective antagonism of adenosine A2A receptors reduces transmitter outflow. Neurobiol Dis.

[75] Liu YF (1998). Expression of polyglutamine-expanded huntingtin activates the SEK1-JNK pathway and induces apoptosis in a hippocampal neuronal cell line. J Biol Chem.

[76] Apostol BL, Illes K, Pallos J (2006). Mutant huntingtin alters MAPK signaling pathways in PC12 and striatal cells: ERK1/2 protects against mutant huntingtin-associated toxicity. Hum Mol Genet.

[77] Morfini GA, You Y-M, Pollema SL (2009). Pathogenic huntingtin inhibits fast axonal transport by activating JNK3 and phosphorylating kinesin. Nat Neurosci.

[78] Fan J, Gladding CM, Wang L (2012). P38 MAPK is involved in enhanced NMDA receptor-dependent excitotoxicity in YAC transgenic mouse model of Huntington disease. Neurobiol Dis.

[79] Ferrante RJ, Kubilus JK, Lee J (2003). Histone deacetylase inhibition by sodium butyrate chemotherapy ameliorates the neurodegenerative phenotype in Huntington's disease mice. J Neurosci.

[80] Taylor DM, Moser R, Régulier E (2013). MAP kinase phosphatase 1 (MKP-1/DUSP1) is neuroprotective in Huntington's disease via additive effects of JNK and p38 inhibition. J Neurosci.

[81] Apostol BL, Simmons DA, Zuccato C (2008). CEP-1347 reduces mutant huntingtin-associated neurotoxicity and restores BDNF levels in R6/2 mice. Mol Cell Neurosci.

[82] Zuccato C, Marullo M, Conforti P, MacDonald ME, Tartari M, Cattaneo E (2008). Systematic assessment of BDNF and its receptor levels in human cortices affected by Huntington's disease. Brain Pathol.

[83] Zuccato C, Ciammola A, Rigamonti D (2001). Loss of huntingtin-mediated BDNF gene transcription in Huntington's disease. Science.

[84] Gauthier LR, Charrin BC, Borrell-Pagès M (2004). Huntingtin controls neurotrophic support and survival of neurons by enhancing BDNF vesicular transport along microtubules. Cell.

[85] Kells AP, Fong DM, Dragunow M, During MJ, Young D, Connor B (2004). AAV-mediated gene delivery of BDNF or GDNF is neuroprotective in a model of Huntington disease. Mol Ther.

[86] Pineda JR, Rubio N, Akerud P (2006). Neuroprotection by GDNF-secreting stem cells in a Huntington's disease model: optical neuroimage tracking of brain-grafted cells. Gene Ther.

[87] Marks WJ, Bartus RT, Siffert J (2010). Gene delivery of AAV2-neurturin for Parkinson's disease: a double-blind, randomised, controlled trial. Lancet Neurol.

[88] Bartus RT, Weinberg MS, Samulski RJ (2014). Parkinson's disease gene therapy: success by design meets failure by efficacy. Mol Ther.

[89] Jiang M, Peng Q, Liu X (2013). Small-molecule TrkB receptor agonists improve motor function and extend survival in a mouse model of Huntington's disease. Hum Mol Genet.

[90] Simmons DA, Belichenko NP, Yang T (2013). A small molecule TrkB ligand reduces motor impairment and neuropathology in R6/2 and BACHD mouse models of Huntington's disease. J Neurosci.

[91] Todd D, Gowers I, Dowler SJ (2014). A monoclonal antibody TrkB receptor agonist as a potential therapeutic for Huntington's disease. PloS One.

[92] Conforti P, Zuccato C, Gaudenzi G (2013). Binding of the repressor complex REST-mSIN3b by small molecules restores neuronal gene transcription in Huntington's disease models. J Neurochem.

[93] Borrell-Pages M, Canals JM, Cordelieres FP (2006). Cystamine and cysteamine increase brain levels of BDNF in Huntington disease via HSJ1b and transglutaminase. J Clin Invest.

[94] (2014). Raptor Pharmaceuticals. Raptor announces clinical results with RP103 in Huntington's disease phase 2/3 trial.

[95] Björkqvist M, Wild EJ, Thiele J (2008). A novel pathogenic pathway of immune activation detectable before clinical onset in Huntington's disease. J Exp Med.

[96] Schwarcz R, Whetsell W, Mangano R (1983). Quinolinic acid: an endogenous metabolite that produces axon-sparing lesions in rat brain. Science.

[97] Vecsei L, Szalardy L, Fulop F, Toldi J (2013). Kynurenines in the CNS: recent advances and new questions. Nat Rev Drug Disc.

[98] Guidetti P, Luthi-Carter RE, Augood SJ, Schwarcz R (2004). Neostriatal and cortical quinolinate levels are increased in early grade Huntington's disease. Neurobiol Dis.

[99] Beal MF, Matson WR, Storey E (1992). Kynurenic acid concentrations are reduced in Huntington's disease cerebral cortex. J Neurol Sci.

[100] Giorgini F, Guidetti P, Nguyen Q, Bennett SC, Muchowski PJ (2005). A genomic screen in yeast implicates kynurenine 3-monooxygenase as a therapeutic target for Huntington disease. Nat Genet.

[101] Campesan S, Green EW, Breda C (2011). The kynurenine pathway modulates neurodegeneration in a Drosophila model of Huntington's disease. Curr Biol.

[102] Zwilling D, Huang SY, Sathyasaikumar KV (2011). Kynurenine 3-monooxygenase inhibition in blood ameliorates neurodegeneration. Cell.

[103] Mrzljak L (2013). Development of kynurenine monooxygenase (KMO) inhibitor. CHDI-340246 for the treatment of Huntington's disease: a progress update.

[104] Traeger U, Andre R, Lahiri N (2014). HTT-lowering reverses Huntington's disease immune dysfunction caused by NFκB-pathway dysregulation. Brain.

[105] Brück W, Pförtner R, Pham T (2012). Reduced astrocytic NF-κB activation by laquinimod protects from cuprizone-induced demyelination. Acta Neuropathol (Berl).

[106] Aharoni R, Saada R, Eilam R, Hayardeny L, Sela M, Arnon R (2012). Oral treatment with laquinimod augments regulatory T-cells and brain-derived neurotrophic factor expression and reduces injury in the CNS of mice with experimental autoimmune encephalomyelitis. J Neuroimmunol.

[107] Mishra MK, Wang J, Silva C, Mack M, Yong VW (2012). Kinetics of proinflammatory monocytes in a model of multiple sclerosis and its perturbation by laquinimod. Am J Pathol.

[108] Comi G, Jeffery D, Kappos L (2012). Placebo-controlled trial of oral laquinimod for multiple sclerosis. N Engl J Med.

[109] Bouchard J, Truong J, Bouchard K (2012). Cannabinoid receptor 2 signaling in peripheral immune cells modulates disease onset and severity in mouse models of Huntington's disease. J Neurosci.

[110] Díaz-Hernández M, Díez-Zaera M, Sánchez-Nogueiro J (2009). Altered P2X7-receptor level and function in mouse models of Huntington's disease and therapeutic efficacy of antagonist administration. FASEB J.

[111] Jun D-J, Kim J, Jung S-Y (2007). Extracellular ATP mediates necrotic cell swelling in SN4741 dopaminergic neurons through P2X7 receptors. J Biol Chem.

[112] Kim K, Lee S-G, Kegelman TP (2011). Role of excitatory amino acid transporter-2 (EAAT2) and glutamate in neurodegeneration: opportunities for developing novel therapeutics. J Cell Physiol.

[113] Liévens JC, Woodman B, Mahal A (2001). Impaired glutamate uptake in the R6 Huntington's disease transgenic mice. Neurobiol Dis.

[114] Arzberger T, Krampfl K, Leimgruber S, Weindl A (1997). Changes of NMDA receptor subunit (NR1, NR2B) and glutamate transporter (GLT1) mRNA expression in Huntington's disease: an in situ hybridization study. J Neuropathol Exp Neurol.

[115] Miller BR, Dorner JL, Shou M (2008). Up-regulation of GLT1 expression increases glutamate uptake and attenuates the Huntington's disease phenotype in the R6/2 mouse. Neuroscience.

[116] Rothstein JD, Patel S, Regan MR (2005). Beta-lactam antibiotics offer neuroprotection by increasing glutamate transporter expression. Nature.

[117] Mrzljak L, Munoz-Sanjuan I (2011). Therapeutic strategies for Huntington's disease. Brain Res.

[118] Seong IS, Ivanova E, Lee J-M (2005). HD CAG repeat implicates a dominant property of huntingtin in mitochondrial energy metabolism. Hum Mol Genet.

[119] Jin J, Albertz J, Guo Z (2013). Neuroprotective effects of PPAR-γ agonist rosiglitazone in N171-82Q mouse model of Huntington's disease. J Neurochem.

[120] Beconi MG, Yates D, Lyons K (2012). Metabolism and Pharmacokinetics of JM6 in Mice: JM6 Is Not a Prodrug for Ro-61-8048. Drug Metabolism and Disposition.

